# Metropolitan Hospital Sunday Fund

**Published:** 1913-02-15

**Authors:** 


### METROPOLITAN HOSPITAL SUNDAY FUND.

### SPECIAL MEETING OF THE CONSTITUENTS.

A very largely attended meeting of constituents of this Fund was held at the Mansion House, under the presidency of the Lord Mayor, on Monday, 10th inst., to consider motions affecting the constitution of the Fund to be moved by the Rev. L. S. Lewis, St. Mark, Whitechapel.

The notice convening the meeting having been read—in the absence of Sir Edmund Hay Currie—by his deputy, letters of regret for absence were read from a number of gentlemen, including his Eminence the Cardinal Archbishop, Canon MacCormac, Rev. Canon Pearce, and Lord Sandhurst.

The Lord Mayor then called upon the Rev. L. S. Lewis to speak to his motions, and asked him to compass his remarks within ten minutes if possible, and subsequent speakers he hoped would not exceed half that time.

The Rev. L. S. Lewis, who was cordially received, first expressed his gratification at the fact that, though he had taken no steps towards it since the last meeting of the Fund in December, this special meeting had been convened by the Council. He recalled to the memory of the meeting the events spoken to at the December meeting owing to a notice of motion which he sent in December 1911 having arrived at the office of the Fund while the Secretary was away ill and been lost. He declared that what he was now bringing forward in the way of resolutions was not of necessity controversial, and he hoped it would remain of that character. No one who was present at the December meeting referred to would doubt that there was a strong feeling that the motions on the subject should be heard. He wished it clearly understood that he had nothing to do with any subject outside the sphere of his own motions. He wished simply to appeal to his brother constituents as to whether they did or did not think the interests of the Fund demanded that certain alterations should be made. Law 1, giving the constituents a voice in the management of the Fund, was not carried out, because there were not sufficient arrangements to carry that out. If the drains of his house were out of order he would not care how the fact was discovered—whether by dead rats discovered by a child playing on the floor, or by a visit of the sanitary inspector; the great point was that they were wrong and must be put right. And there was something wrong in the constitution of this Fund, though he would not waste the time of the meeting by detailing how it was discovered. Constituents, during a course of years, tried to get certain alterations and to have certain things referred back, and they discovered that the powers granted by the constitution were practically useless. What he proposed that day was only a patch in the effort to put right what at present was only an intolerable farce; the whole of the constitution required recasting. There was at present nothing to show how people were to be nominated to a seat on the Council, nor how to call a special meeting, nor as to the length of notice which must be given; all that kind of thing was left to haphazard. There was not always the same chairman at the meetings of the Fund, and there were very few people who understood the business of taking the chair, even when they had the best intentions; they often seemed to think it was their function to legislate instead of to judge, to interpret the laws which had been already laid down. He had more than once called for—as he did at the meeting last December—the particular law which governed the decision just given, but it was not stated. But he had not time on the present occasion to deal with the whole constitution. He would recall that at the meeting in December 1911, when the Lord Mayor was in the chair and the Bishop of London was present, an appeal was made to him to withdraw the motion he had brought, and he did so on the distinct understanding that the whole matter would be thrashed out. The Lord Mayor undertook personally to make inquiry, and he went to a certain hospital and made inquiry. But, of course, he (Mr. Lewis) did not withdraw his motion so that one person should make an inquiry about one hospital; but the idea was for the whole matter to be gone into. Sir Henry Burdett, whose authority on hospital matters was probably second to none, had supported this claim for the revision of the constitution of the Fund. It would be considered an impertinence of him to propose a revision of the whole constitution, yet matters had a habit of concentrating themselves when a nucleus was established. He would deprecate any aspersion whatever on Sir Edmund Currie, whose great work for the Fund and uniform courtesy he praised in the highest manner; the lapse which had occurred in this matter, during illness, might have happened to anybody. He proceeded to read the words from The Hospital of December 28, 1912, in comment of the report of the last meeting of constituents, deploring the absence of proper by-laws for regulating the proceedings of the Fund and requiring a more real control on the part of the constituents, especially in view of the growing restiveness of the latter. He (Mr. Lewis) did not wish to stand absolutely for all the wording which he had brought forward. He was willing to compromise on some. He asked to be allowed to take the second resolution before the first. This the meeting allowed, on the proposition of the Hon. Sydney Holland. Mr. Lewis therefore moved: "That Law 10 of the constitution shall be changed to the following: 10. The Committee of Distribution shall present their report to the Council for approval, and before the Fund shall be distributed the Council's recommendations shall be confirmed by the annual meeting. In the event of any award being rejected at the annual meeting, an Arbitration Committee, whose decision shall be final, shall be appointed at that meeting to deal with that particular point, such Arbitration Committee to consist of three members elected by the constituents of the Fund at that meeting, three members elected by the Council, and a chairman chosen from outside the Fund, to be agreed upon by the other six members of the Arbitration Committee." His object, he said, was to end the prolonged bickerings and discussions which had been going on. The important people in the Fund were the Distribution Committee. The latter reported to the Council in July or August, the Council confirmed, then, in the month of December, the constituents were called together to elect the Council for the succeeding year and to receive the annual report. More than once the constituents had objected to something and moved that it be referred back. But the reply to that was that the money had been paid over in the previous August. Owing to St. George's Hospital not agreeing to keep its accounts on the uniform system adopted by other hospitals, and which permitted comparisons to be made among various hospitals, the King's Fund granted that hospital in 1909 only £188. The hospital in question went behind the King's Fund and obtained from the Sunday Fund a grant of £1,545. When that particular grant was moved to be referred back the answer given was that it was of no use as the money was handed over last August. That he declared to be a complete farce; it insulted the constituents, who ought not to be called together to be mocked at in that way. One of his desires was to put that right. It would be impossible to adequately deal with details in a great meeting such as this, therefore he had tried to propose that they should form themselves into a sort of grand jury to see whether or not there was a *prima facie* case. So that if in any year the constituents thought any grant ought to be referred back, then under the machinery he was proposing an Arbitration Committee would be appointed, whose decision should be final.

This was duly seconded.

The Hon. Sydney Holland, in opposing, said he feared he could not agree with Mr. Lewis that the matter was not controversial. That gentleman had said that if the drains in his house were wrong he would not mind how or by whom it was discovered, whether by dead rats being found or by the visit of the sanitary inspector. But he (Mr. Holland) would first want to be sure that the drains were wrong. Mr. Lewis had pointed out certain parts of the constitution which he said were wrong, but there was nothing in Mr. Lewis's proposition to set them right. Therefore the Council of the Fund had appointed a sub-committee to draw up a new set of rules for the management of the Fund on a matter of detail. The constitution was drawn up many years ago, when the Fund started, when it had not so many critics as there were to-day. Under its constitution the best possible had been done. He would not enter into the question of the St. George's Hospital grant, as that was at present *sub judice*, except to say that that hospital had a stronger case from their point of view than many people thought. He reminded the meeting that at present the Council of the Fund was elected by the constituents, and if the latter did not like the Council they could elect a fresh one, just as discontented shareholders could have a new board of directors. By the Council were elected that most important body, the Distribution Committee, chosen from men who were experts in hospital management and well-known business men in the City, and who were not partisans in any one hospital, and they tried to exclude any men who had a "bee in their bonnet." This Distribution Committee were about as strong a body as could be got together. They sat for hours hearing and discussing complaints and claims from various hospitals, and then the general Council went through their recommendations to see if they all appeared fair. So pleased was the late George Herring with the management of this Fund—and he was himself a well-known man of business—that he left to this Fund £600,000 rather than to any of the others. The decisions of this Distribution Committee had, until Mr. Lewis and Mr. Stephen Coleridge came upon the scene, been received with acclamation, and with some gratitude to the gentlemen who had devoted so much time to this question. He personally had no reason to be pleased with the verdicts of the Distribution Committee, but they would not put him on because he was an advocate for two, if not three, hospitals, and they had given decisions which he regarded as unfair to his own special hospital. But in everything one must accept the verdict of the umpire; and if he knew the decision was arrived at after careful consideration he concluded there was a possibility that they were right and he was wrong. But what were they to do now? He thought he ought really to second Mr. Lewis's motion, for he could see a very nice vision before him. He had the largest number of contributors to the London Hospital, and he could see a way of packing a meeting as a means of getting a much larger grant for that hospital than at present. In future, it would seem, the report of the Distribution Committee was to be cut about, and partisans were to fight for their own particular hospital. Mr. Lewis said it must be referred to arbitration, but if he (Mr. Holland) was strong enough to pack a meeting so as to get an award referred back to arbitration, he would be strong enough to see that three of his supporters were elected on the Arbitration Committee, and so he would have half the Arbitration Committee on his side.

Mr. Lewis here rose to protest that Mr. Holland was misrepresenting him and the purpose of his motion. Nothing was to be referred to arbitration unless a strong feeling was manifested against any award or decision.

Mr. Holland replied that it would be quite easy for the hypothetical meeting, consisting of his friends, to move that all the awards should be referred back; or he could move that the one to the London Hospital be referred back, with the idea of getting it doubled. Mr. Lewis had declared that he brought forward the motions in the interests of the Fund. He must forgive him for doubting that. He reminded his hearers of the teetotal lecturer who, getting husky, was given a glass of water and proceeded to blow the froth off, and said he preferred rather to judge Mr. Lewis by what he did outside than by what he said at those meetings. At the time Mr. Lewis went to his present church the collections for the Fund were £2 13s., but during Mr. Lewis's incumbency the record was reached last year with 8s. 11d., the amount for the first year being 3s. 3d. He had good reason to know that East End parishes were poor, but others among them gave ten and twenty times as much to the Fund as did Mr. Lewis's. It really meant that Mr. Lewis came to the meetings of the Fund as the mouthpiece of the Anti-vivisectionists, who thought the Fund was unfair in not giving an award to the Anti-Vivisection Hospital.

Rev. Mr. Sturges (St. Barnabas, South Lambeth), in support of Mr. Holland's contention, said he hoped none of those present would give a vote which might upset the work of the Fund. Until six years ago there had been no discord, and that such occurred now was due to a party of cranks, who had the idea that they had only to plead about cruelty to secure that every clergyman would fall round their necks and do everything they asked. He did not see where the injustice to subscribers came in. He wanted the meeting to clearly see that the opposition originated in the anti-vivisection crusade, and nine out of every ten persons who spoke on that subject did not understand it, and, indeed, the term itself was a misnomer.

Mr. Stowe (St. Mary, Whitechapel) spoke in strong support of Mr. Lewis, desiring that the constituents should possess in the Fund the power which they were declared to have in theory. He objected to people who urged the alteration having personalities hurled at them, and the reference to the small collections at Mr. Lewis's church was in very bad taste. There was dissatisfaction in connection with the Fund, whose income had gone down of late, whereas that of the King's Fund had risen in the same period.

Sir Savile Crossley spoke in support of the position taken up by Mr. Sydney Holland on this matter, as it seemed to him sound. The reason given by Mr. Lewis for the King's Fund withholding a grant from a certain hospital was not quite correct, and he asked the meeting to suspend its judgment on that matter. He repeated that a committee had been appointed to go into the necessary reforms, which was an earnest that they wished to do the best possible for the Fund.

Mr. Lewis wished to defend his poor parish from the aspersion which had been cast upon it. It consisted of 6,500 foreign Jews out of a total of 7,500, and in it was a Roman Catholic colony, while many of the parishioners were casual labourers. His predecessor was a wealthy man, and he himself contributed to the Fund. He deplored the fact that the gifts of the poor had been ridiculed. Any falling-off was due to a feeling of mistrust of the Fund. If the constituents were content to be called together in December to approve what had been done in August, and to be deprived of power to refer back any award they objected to, they were the only assembly in Europe so easily pleased, and he was amazed.

The resolution was then put, and lost by a large majority.

Mr. Lewis then, amid cries of "Withdraw!" moved the next resolution: "That Law 3 of the Constitution be changed to the following: 3. The Committee of Distribution shall consist of the Lord Mayor, as President, and the Hon. Secretaries, *ex officio*, and nine other members, of whom four only shall be doctors or surgeons, who shall be elected at the annual general meeting."

Mr. Counsel seconded, and it was lost.

Mr. Lewis then moved his third resolution, as follows: "That in Law 1 of the Constitution the words 'in the month of December' be deleted."

Mr. Counsel, in seconding, said one question which might arise at a month's or even a week's notice was, how did one know that hospitals receiving money from the congregations might not refuse to give the help needed, on the ground that the applicants were insured under the Act?

Mr. Holland replied that out of 600 applicants at the London Hospital who were insured, only thirty-four had been refused, and that was simply because they were "chronics."

This resolution was also lost, and a vote of thanks to the Lord Mayor for his services in the chair concluded a very animated meeting.

